# A Novel Clinical Nomogram for Predicting Unfavorable Tuberculosis Treatment Outcomes: A Logistic Regression Risk Model

**DOI:** 10.1007/s44197-026-00532-z

**Published:** 2026-03-18

**Authors:** Sancho Pedro Xavier, Gelcídio Alfredo Pereira Rafael, Ana Raquel Manuel Ernesto Gotine, Mateus António Agostinho, Graciano Cumaquela, Zito António Joaquim Rocha, Audêncio Victor

**Affiliations:** 1 Public Health Postgraduate Program, School of Public Health, University of São Paulo (USP), Avenida Doutor Arnaldo, 715, São Paulo, São Paulo 01246904 Brazil; 2 Nacaroa District Services for Health, Women and Social Action, Nampula, Mozambique; 3 Rovuma University, Nampula, Mozambique; 4 Faculty of Health Sciences, Lúrio University, Nampula, Mozambique; 5 Faculty of Epidemiology and Population Health, London School of Hygiene & Tropical Medicine, Keppel Street, London, WC1E 7HT UK

**Keywords:** Tuberculosis, Treatment outcomes, Unfavorable outcome, Nomogram, Predictive model, Mozambique

## Abstract

**Introduction:**

Communicable diseases remain one of the major public health challenges in Sub-Saharan Africa, with tuberculosis (TB) ranking among the leading causes of morbidity, mortality, and significant economic impact. Mozambique is among the countries with the highest TB burden in the region. This study aimed to develop a clinical prediction model, in the form of a nomogram, to predict the probability of unfavorable treatment outcomes (UTO) among TB patients treated at a district health center in Nacarôa, Nampula Province, Mozambique.

**Methods:**

A retrospective cohort study was conducted using secondary data from patients diagnosed and treated for TB between 2021 and 2023. A multivariable logistic regression analysis was performed to identify factors associated with UTO, and a predictive nomogram was subsequently constructed. Model performance was assessed using the receiver operating characteristic (ROC) curve, accuracy, Brier Score (BS), calibration plot, and the Hosmer–Lemeshow goodness-of-fit test. Clinical utility was evaluated through decision curve analysis (DCA) and clinical impact curves.

**Results:**

UTO were observed in 26.8% of patients (55/205). The multivariable analysis identified as significant predictors of UTO being previously treated for TB, not receiving directly observed therapy (DOT), having a clinical or radiological diagnosis, and having a positive smear microscopy result. The nomogram showed good performance, with an AUC of 83.2% and an accuracy of 84.9%. The Hosmer–Lemeshow test indicated good model fit (*p* = 0.132), and the calibration plot demonstrated strong agreement between predicted and observed outcomes (BS = 0.119). DCA and clinical impact analyses confirmed the model’s potential to support and optimize clinical decision-making in TB management.

**Conclusion:**

The nomogram developed in this study represents a promising and practical tool for estimating the individual risk of UTO in tuberculosis care and may contribute to improved clinical management and resource allocation in high-burden settings.

**Supplementary Information:**

The online version contains supplementary material available at 10.1007/s44197-026-00532-z.

## Introduction

Tuberculosis (TB), despite being considered an ancient disease, remains one of the most lethal infectious diseases worldwide, particularly in low- and middle-income countries [1, 2]. It is estimated that 8.2 million people were diagnosed with tuberculosis worldwide, marking an increase compared to 7.5 million in 2022 and 7.1 million in 2019 [3]. These numbers were significantly higher than the 5.8 million and 6.4 million cases reported in 2020 and 2021, respectively, likely due to the disruptions caused by the COVID-19 pandemic, which led to delays in diagnosis and treatment [4]. In Mozambique, the TB incidence rate is 361 cases per 100,000 population, resulting in an estimated 8,000 deaths [5].

Despite the availability of effective treatment, there remains a significant number of unfavorable outcomes, such as treatment abandonment, relapse, and death that continue to challenge TB control programs [6, 7]. These adverse TB outcomes are often associated with multiple clinical and social factors, including age, sex, smoking, alcohol abuse, diabetes, HIV infection, and a history of incomplete or non-adherent treatment [8, 9]. The complexity of these factors highlights the need for more accurate predictive tools capable of identifying, early in the course of treatment, patients at higher risk of poor outcomes [8].

Multivariable statistical models, such as logistic regression, have been widely used to predict clinical outcomes in various fields of medicine [10]. One practical application of these models is the development of nomograms, a graphical tools that translate statistical equations into easy-to-interpret visual scales. These tools allow for individualized risk estimation of clinical events and can assist physicians in making personalized predictions for each patient based on known risk factors, thereby improving patient prognosis if appropriate interventions are taken in time [11–13].

Recent studies have shown that nomogram models are widely used to establish clinical prognostic models for TB and multidrug-resistant TB in Asian settings [8, 14, 15]. In Ethiopia, studies have shown that the nomogram model was considered useful in clinical practice and is clinically interpretable [16]. Therefore, this study aims to develop and validate a clinical nomogram to predict the probability of unfavorable treatment outcomes (UTO) among TB patients treated at the health center in Nacarôa, Nampula Province, Mozambique.

## Methods

### Study Design and Setting

A retrospective cohort study was conducted using data from the National Tuberculosis Control Program (NTCP), based on registry books and treatment records of patients treated at the Nacarôa Health Center (HC), a referral unit for tuberculosis care in the district. The Nacarôa HC is located in Nacarôa District, Nampula Province, Mozambique. According to the 2007 Census, the district had an estimated population of 106,887 inhabitants, distributed over an area of 2,793 km², resulting in a population density of approximately 38.27 inhabitants per km². Data were collected between February and March 2024.

### Population and Sample Size

Patients diagnosed and treated for TB with records in the NTCP between 2021 and 2023 were included. During this period, 453 patients were reported. One transferred patient, 28 without treatment outcome records, and four with multidrug-resistant tuberculosis were excluded, resulting in 420 eligible patients. The minimum sample size was calculated as 191 patients, considering a 5% margin of error (d = 0.05), a 95% confidence level (Z = 1.96), and an estimated proportion of 35%, based on previous studies reporting rates of up to 33.5% [17, 18]. The sample size was calculated using Epi Info software, version 7 [19, 20]. The formula used is shown below and corresponds to the Cochran equation adjusted for finite populations.

$$n=\frac{p(1-p){Z}^{2}N}{{\mathrm{d}}^{2}\left(N-1\right)+{Z}^{2}p(1-p)}$$

To improve the representativeness and precision of the estimates, 205 patients were included in the study. Participants were selected through systematic random sampling from the eligible population. To obtain the sample of 205 patients, the selection interval (K) was calculated by dividing the total number of eligible patients (N) by the desired sample size (n): K = N/*n* = 420/205 ≈ 2.05. Patient identification numbers (IDs) were listed, and a random draw determined the starting point (number 2). From that point, every second patient (2, 4, 6, 8…) was selected until the target sample size of 205 was reached.

### Study Variables

The outcome variable was the favorability of the treatment outcome, classified as favorable or unfavorable, according to World Health Organization (WHO) guidelines. A favorable outcome was defined as the sum of cured and treatment completed. Cure was assigned to patients with pulmonary tuberculosis and bacteriological confirmation at treatment initiation who achieved negative smear or culture results at the end of treatment and on at least one previous occasion. Treatment completion referred to patients who finished the full therapeutic regimen with no clinical, radiological, or operational evidence of treatment failure, regardless of laboratory confirmation of negativity [21]. Patients with unfavorable outcomes included those who abandoned treatment, experienced treatment failure, died, or were not evaluated [21, 22].

Independent variables included age (< 15 and ≥ 15 years), place of residence, disease category according to WHO (with new cases defined as patients who have never been treated for TB or have received anti-TB medication for less than one month, and previously treated patients defined as those who have received one month or more of anti-TB drugs in the past, which includes relapse cases, treatment after failure, treatment after loss to follow-up, and other previously treated patients [21]; treatment phases, classified into two groups: intensive phase (two months) and continuation phase (four or six months) [24]; HIV serostatus; implementation of directly observed treatment (DOT), defined as receiving the entire course of treatment under direct observation, supervised by healthcare personnel or community volunteers; drug regimen for adults and children weighing ≥ 25 kg, and for children weighing < 25 kg; type of diagnostic methods; bacteriological (smear microscopy) categorized as yes/no; positive smear at the 2nd and 5th month of treatment; total treatment duration; and occurrence of adverse events during therapy, which were routinely recorded by trained healthcare technicians working in the TB program, as detailed in Table 1.

**Table 1:** Baseline characteristics of patients included in the tuberculosis treatment outcome cohort

Variable	Total (*n* = 205)	Unfavorable (*n* = 55)	Favorable (*n* = 150)	*p*-value
Age				
< 15 years	12 (5.9%)	2 (16.7%)	10 (83.3%)	0.629
≥ 15 years	193 (94.1%)	53 (27.5%)	140 (72.5%)	
**Sex**				0.661
Female	89 (43.4%)	22 (24.7%)	67 (75.3%)	
Male	116 (56.6%)	33 (28.4%)	83 (71.6%)	
**Place of residence**				0.228
Inteta	57 (27.8%)	20 (35.1%)	37 (64.9%)	
Nacarôa Sede	114 (55.6%)	28 (24.6%)	86 (75.4%)	
Saua Saua	34 (16.6%)	7 (20.6%)	27 (79.4%)	
**Patient classification (WHO)**				< 0.001
New case	170 (82.9%)	37 (21.8%)	133 (78.2%)	
Previously treated	35 (17.1%)	18 (51.4%)	17 (48.6%)	
**Type of TB**				1.000
Pulmonary	201 (98.0%)	54 (26.9%)	147 (73.1%)	
Extrapulmonary	4 (2.0%)	1 (25.0%)	3 (75.0%)	
**Treatment phase**				< 0.001
Intensive	44 (21.5%)	44 (100.0%)	0 (0.0%)	
Continuation	161 (78.5%)	11 (6.8%)	150 (93.2%)	
**HIV status**				0.480
Negative	172 (83.9%)	44 (25.6%)	128 (74.4%)	
Positive	33 (16.1%)	11 (33.3%)	22 (66.7%)	
**DOT (Directly Observed Therapy)**				< 0.001
Yes	71 (34.6%)	7 (9.9%)	64 (90.1%)	
No	134 (65.4%)	48 (35.8%)	86 (64.2%)	
**Treatment regimen (≥ 25 kg)**				< 0.001
2DF	148 (72.2%)	9 (6.1%)	139 (93.9%)	
4DFC	46 (22.4%)	44 (95.7%)	2 (4.3%)	
Not applicable	11 (5.4%)	2 (18.2%)	9 (81.8%)	
**Treatment regimen (< 25 kg)**				0.008
2DFC	11 (5.4%)	0 (0.0%)	11 (100.0%)	
3DFC	2 (1.0%)	2 (100.0%)	0 (0.0%)	
Not applicable	192 (93.7%)	53 (27.6%)	139 (72.4%)	
**Diagnostic method**				0.003
Bacteriological	169 (82.4%)	53 (31.4%)	116 (68.6%)	
Clinical/X-ray	36 (17.6%)	2 (5.6%)	34 (94.4%)	
**Smear microscopy**				0.140
Yes	90 (43.9%)	19 (21.1%)	71 (78.9%)	
No	115 (56.1%)	36 (31.3%)	79 (68.7%)	
**Smear positivity (2nd month)**				< 0.001
No	120 (58.5%)	20 (16.7%)	100 (83.3%)	
Not applicable	69 (33.7%)	23 (33.3%)	46 (66.7%)	
Yes	16 (7.8%)	12 (75.0%)	4 (25.0%)	
**Smear positivity (5th month)**				< 0.001
No	110 (53.7%)	0 (0.0%)	110 (100.0%)	
Not applicable	81 (39.5%)	42 (51.9%)	39 (48.1%)	
Yes	14 (6.8%)	13 (92.9%)	1 (7.1%)	
**Treatment duration (Median = 6.0; IQR: 5.0–6.0)**				< 0.001
≥ 6 months	147 (71.7%)	1 (0.7%)	146 (99.3%)	
< 6 months	58 (28.3%)	54 (93.1%)	4 (6.9%)	
**Adverse events**				< 0.001
No	193 (94.1%)	44 (22.8%)	149 (77.2%)	
Yes	12 (5.9%)	11 (91.7%)	1 (8.3%)	

### Statistical Analysis

Descriptive analysis was initially performed. Categorical variables were presented as absolute and relative frequencies. Quantitative variables were expressed as mean and standard deviation when normally distributed; otherwise, as median and interquartile range.

Associations between categorical variables were assessed using the chi-square test. Logistic regression was used for both univariable and multivariable analyses. For each variable, the reference category was selected based on clinical relevance and supported by the literature [17, 18]. For example, for the variable Patient category (WHO), “New case” was chosen as the reference group because it represents the standard initial presentation of TB. For DOT, “Yes” was selected as the reference group because it reflects the recommended treatment strategy and is associated with better adherence. Variables with p-values < 0.20 in the univariable analysis were prioritized for inclusion in the multivariable model. Variables showing high collinearity were excluded (**S.** Table 1). Statistically significant variables were retained in the final model. Adjusted odds ratios (AORs) and their corresponding 95% confidence intervals were calculated. A p-value < 0.05 was considered statistically significant.

A predictive model was then developed in the form of a nomogram, based on the factors retained in the multivariable model, and it also incorporated other variables, including adverse drug reactions and second-month smear positivity, which were not included in the final model but were considered important for prediction. The model’s goodness of fit was assessed using the Hosmer–Lemeshow test (*p* > 0.05 indicating good fit). The Likelihood Ratio Test was applied to assess the overall significance of the nomogram.

Model performance was assessed using the calibration curve, the receiver operating characteristic (ROC) curve, the Brier Score (BS), Decision Curve Analysis (DCA), and clinical impact curves. Internal validation was performed using bootstrapping with 1,000 repetitions (boot). The initial classification of the model was based on a threshold of 0.50, and the optimal threshold was subsequently determined using the Youden Index (J) [23]. Performance metrics, including accuracy, sensitivity, specificity, positive predictive value, and negative predictive value, were then calculated.

All data analyses were performed using R software version 4.5.1 (R Project for Statistical Computing, RRID: SCR_001905) and RStudio version 2025.05.1 + 513, released on 2025-06-05.

## Results

### Characteristics of the Study Population

Among the total number of patients included in the study, the majority were over 15 years of age (94.1%), male (56.6%), and residing in Nacarôa Sede (55.6%). The new case of TB was 82.9%, and among these patients, 21.8% experienced UTO. Pulmonary TB was the predominant clinical form, diagnosed in 98% of the patients, with 25% of these cases resulting in UTO (Table 1).

**Fig. 1:**
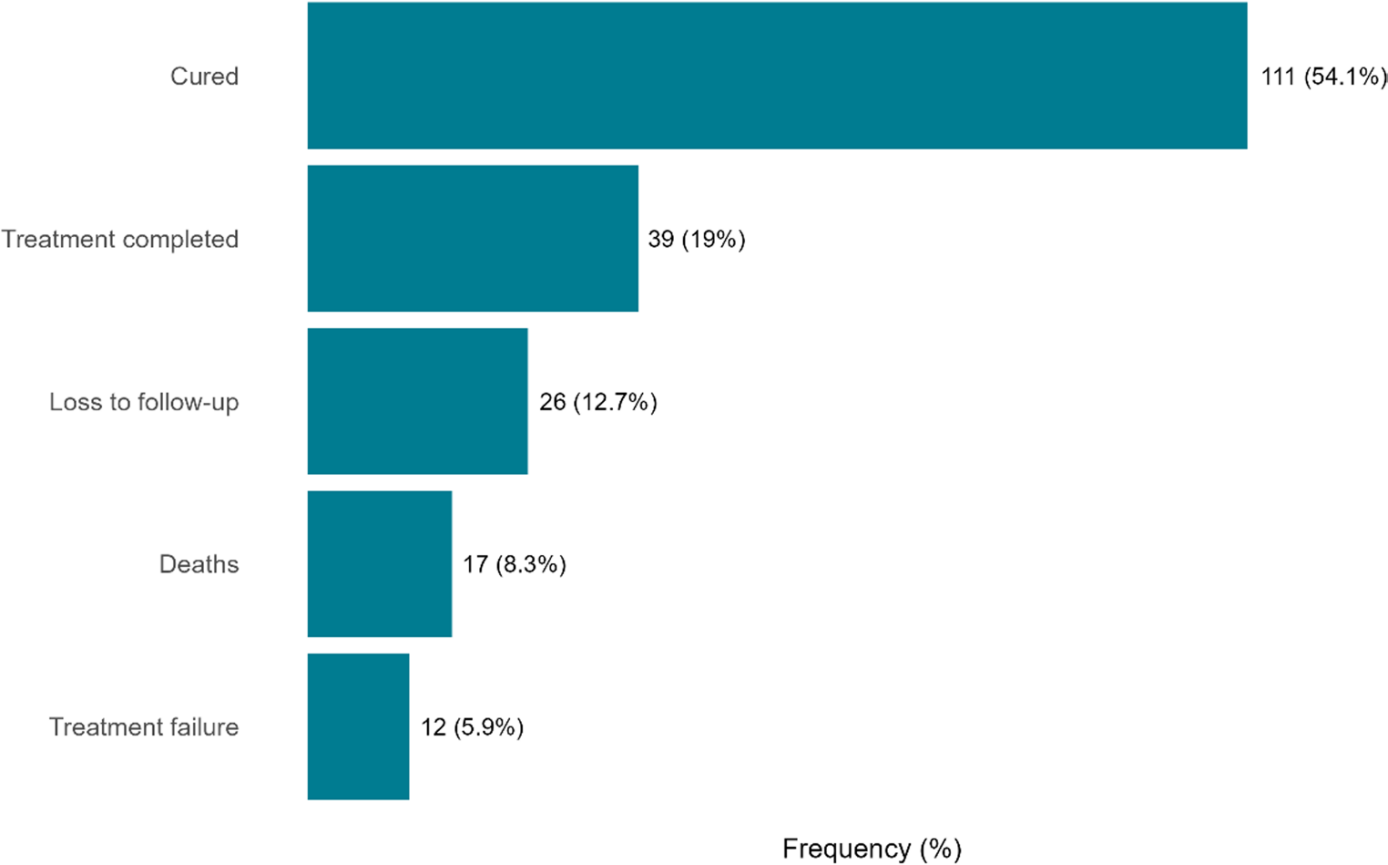
Distribution of tuberculosis treatment outcomes

Overall, 55 patients (26.8%) had UTO, while 150 (73.2%) achieved favorable outcomes. Most patients were classified as cured (53.7%), followed by those who completed treatment (19.5%). Unfavorable outcomes were composed of loss to follow-up (13.7%), death (8.3%), and treatment failure (4.9%) (See S1**.** Figure 1 and Fig. 1).

### Factors Associated with Unfavorable Outcomes

Table 2 presents the predictors of UTO identified in the multivariable logistic regression analysis. Not receiving DOT and having been previously treated for TB were significantly associated with a higher likelihood of UTO. In contrast, a clinical or radiological diagnosis and a positive smear microscopy result were associated with a lower likelihood of UTO compared to bacteriological diagnosis.

**Table 2:** Univariable and multivariable logistic regression analysis of factors associated with UTO

Variables	Univariable model			Multivariable model		
	OR	IC95%	*p*-value	AOR	IC95%	*p*-value
**Patient category (WHO)**						
New case	Reference			Reference		
Previously treated	3.81	1.79; 8.11	< 0.001	3.43	1.47; 8.02	0.005
**DOT**						
Yes	Reference			Reference		
No	5.10	2.17; 12.02	< 0.001	3.82	1.56; 9.34	0.003
**Diagnostic method**						
Bacteriological	Reference			Reference		
Clinical/X-ray	0.13	0.03; 0.56	0.006	0.08	0.02; 0.39	0.001
**Smear microscopy**						
No	Reference			Reference		
Yes	0.59	0.31; 1.12	0.104	0.39	0.19; 0.81	0.011

### Development of the Nomogram Model

**Fig. 2:**
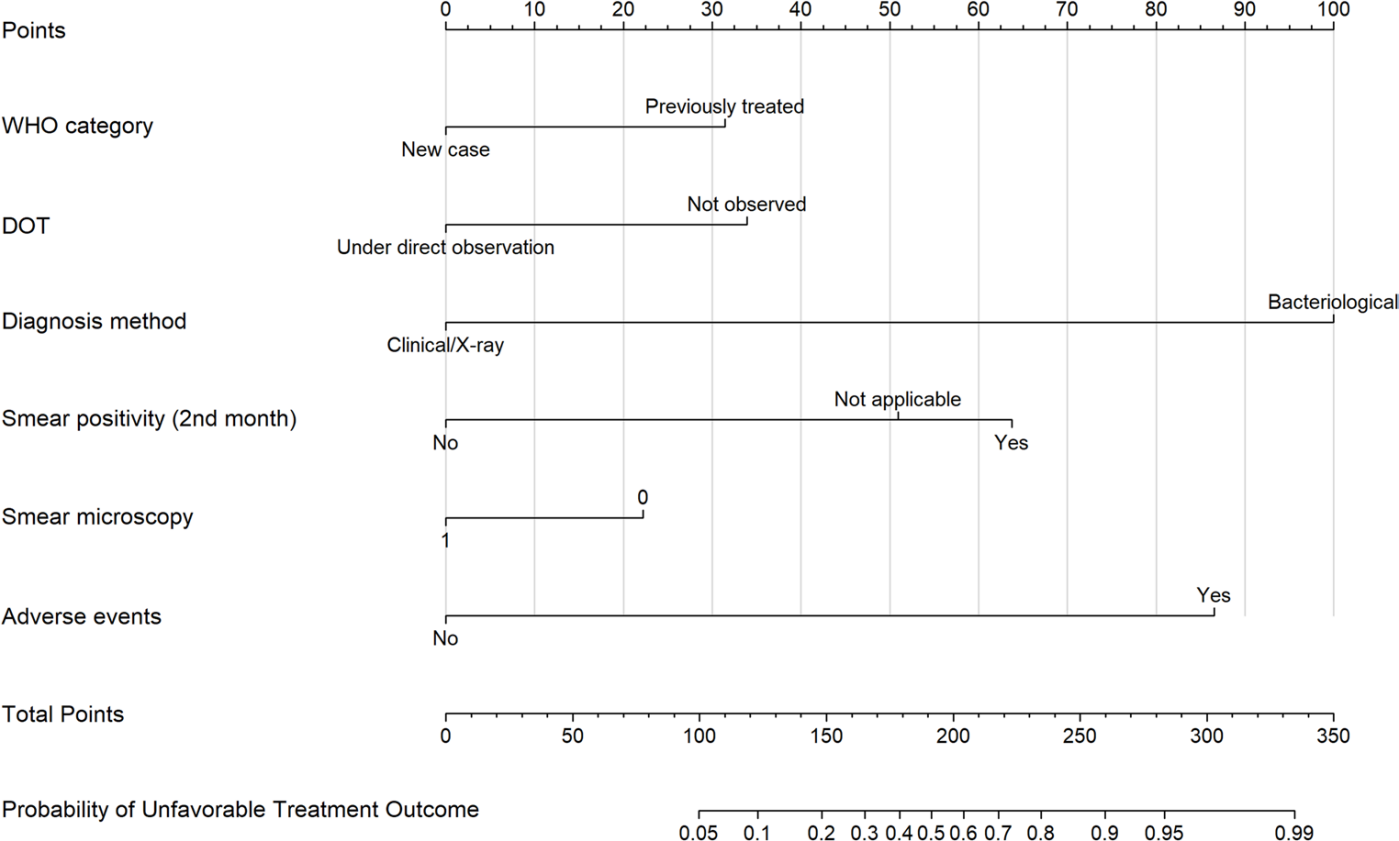
Nomogram for predicting UTO using clinical and programmatic variables

A clinical nomogram was developed to estimate the probability of UTO based on the selected predictors (Fig. 2). The nomogram included six variables, namely WHO TB category, DOT status, diagnostic method, smear result at the second month of treatment, smear microscopy, and the presence of adverse drug reactions. Each variable contributes to a total score that allows individualized risk prediction (S. Table 3). For example, consider a patient who was previously treated for TB, whose DOT was not observed, was diagnosed clinically or by chest X-ray, had a positive smear in the second month of treatment, underwent smear microscopy, and experienced adverse drug reactions. The total nomogram score for this patient is 216, which corresponds to an estimated probability of an UTO of approximately 70%.

### Evaluation and Validation of the Nomogram

**Fig. 3:**
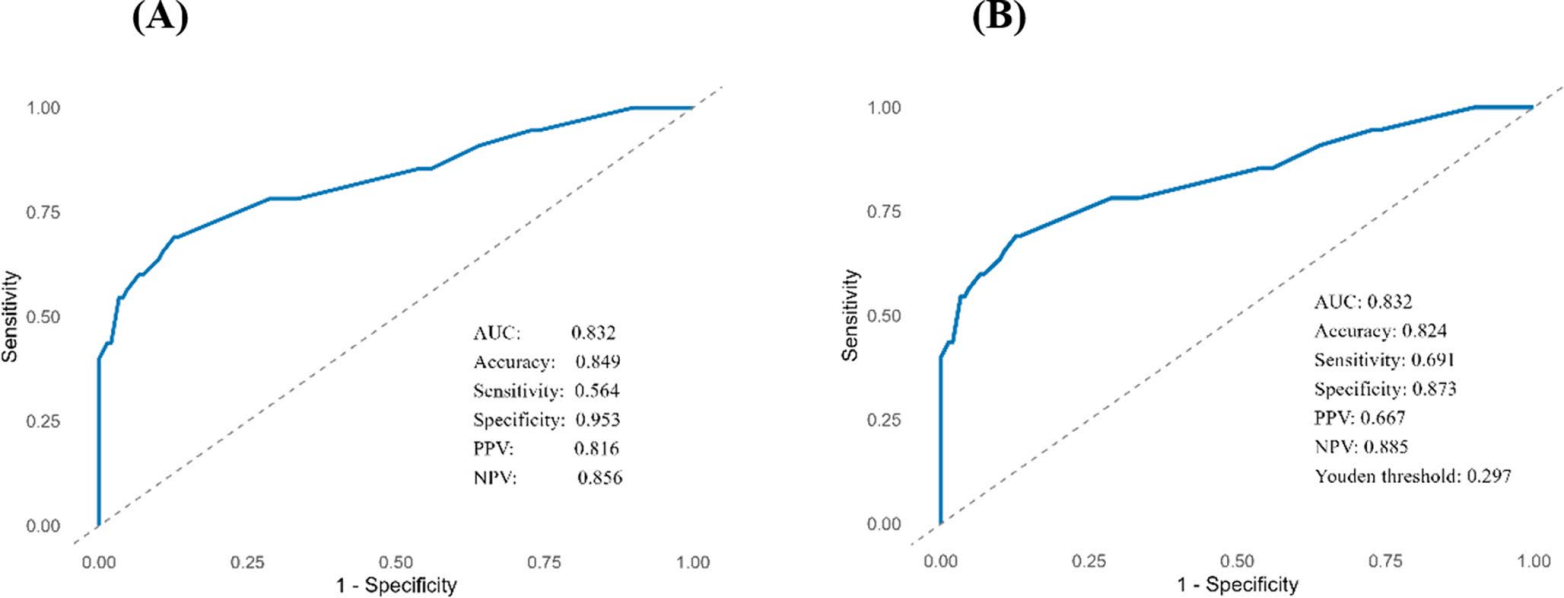
ROC curves assessing the discriminatory performance of the nomogram, with (**A**) a threshold of 0.50 and (**B**) the optimal threshold according to Youden’s index (0.297)

The ROC curve analysis showed good discrimination of the model, with an AUC of 0.832. Using a threshold of 0.50, the nomogram achieved an accuracy of 0.849, with a sensitivity of 0.564, specificity of 0.953, positive predictive value of 0.816, and negative predictive value of 0.856 (Fig. 3). The Youden Index (J) identified 0.297 as the optimal threshold. With this threshold, the accuracy was 0.824, sensitivity 0.691, specificity 0.873, and the positive and negative predictive values were 0.667 and 0.885, respectively (Fig. 3 and S. Table 4).

**Fig. 4:**
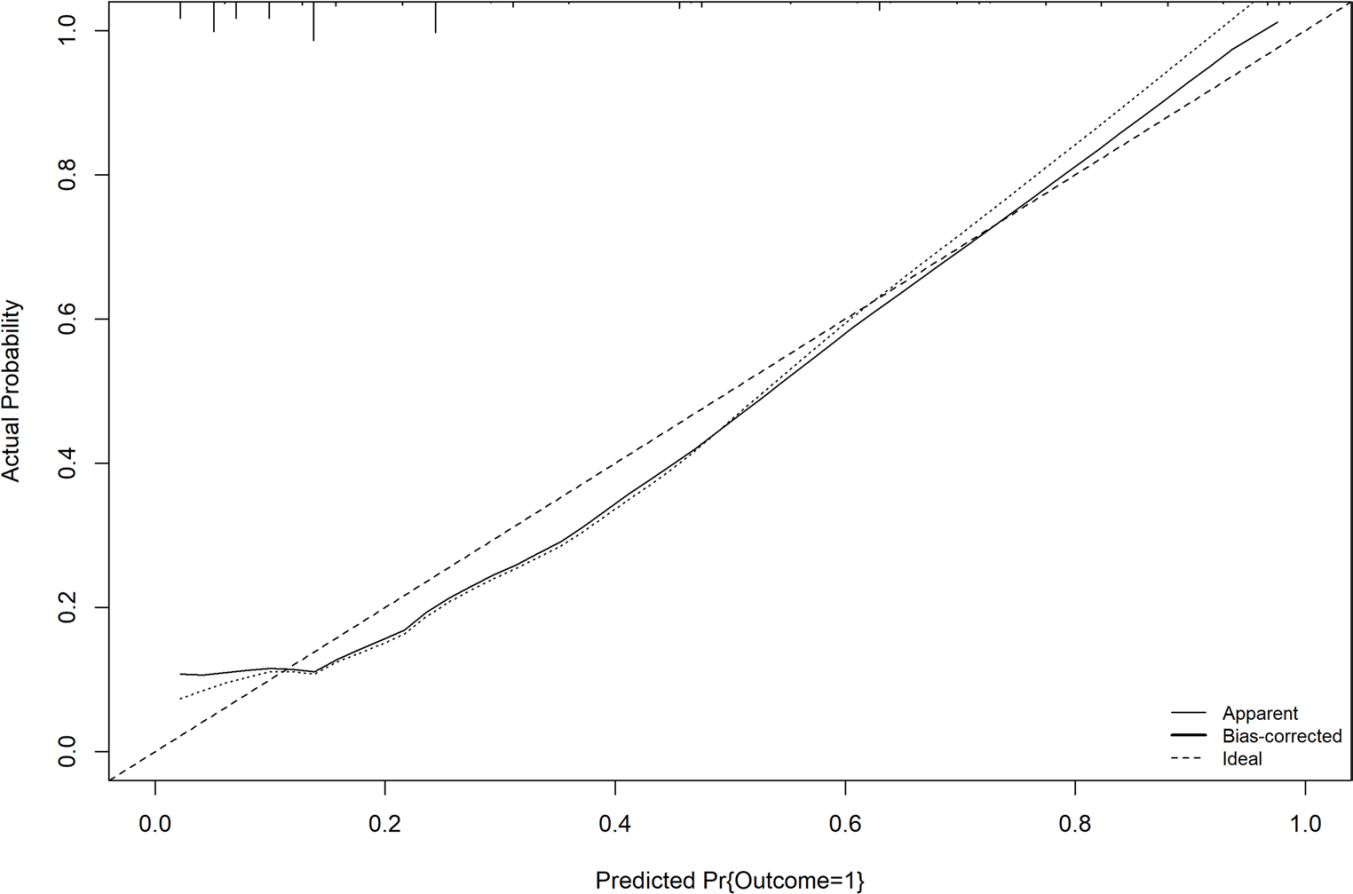
Calibration plot for the predictive nomogram model

The calibration curve showed good agreement between the predicted and observed probabilities, with a Brier Score of 0.119 (Fig. 4 and S. Table 5), and the Hosmer–Lemeshow test confirmed the model’s goodness of fit (χ² = 8.466; *p* = 0.1323) (S**.** Table 2). The Likelihood Ratio Test was statistically significant (LR χ² = 77.61; *p* < 0.001) (S. Table 5). Internal validation was performed using 1,000 bootstrap resamples. The C-index was 0.842 for the training set, 0.826 for the test set, and 0.815 for the optimism-corrected estimate, with a corrected Brier Score of 0.13, indicating good discriminative ability and model stability (S. Tables 6 and 7; S. Figure 2).

### Clinical Utility of the Model

**Fig. 5:**
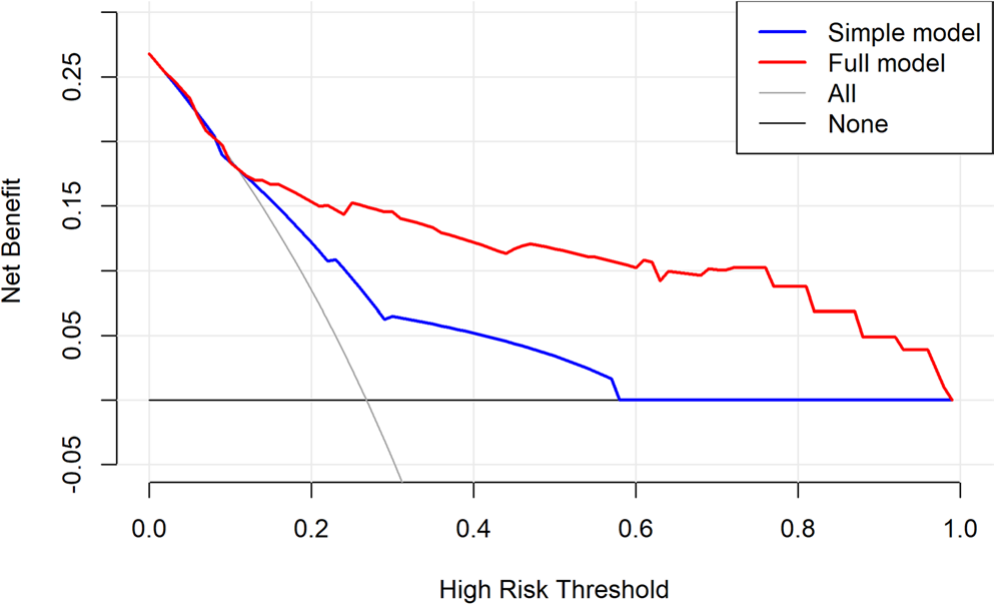
DCA comparing the clinical utility of the simple model, including DOT and patient category (WHO), versus the full model, including DOT, patient category (WHO), diagnosis method, smear positivity (2nd month), smear microscopy, and adverse events **A B**

**Fig. 6:**
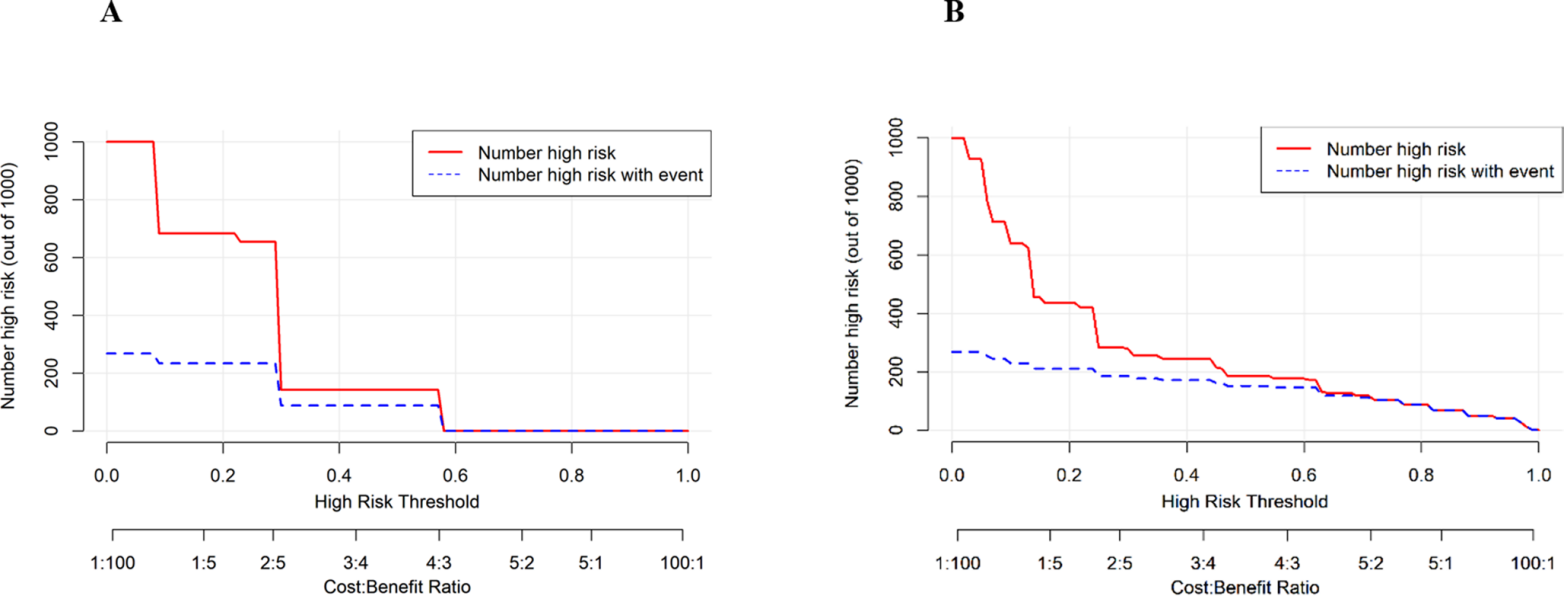
Clinical impact curves for the prediction of UTO: (**A**) Simple model; (**B**) Full model. Red line: number of patients classified as high risk per 1000; Blue dashed line: number of true positives among those classified as high risk. The full model consistently identifies more true positive cases with fewer unnecessary interventions

The DCA demonstrated the clinical usefulness of the nomogram in predicting UTO (Fig. 5). The clinical impact curves (Fig. 6) further supported its applicability, showing a positive net benefit and reinforcing its potential to guide clinical decision-making in TB care.

## Discussion

The occurrence of UTO in this study was 26.8%. This result differs from findings of studies conducted in Ethiopia (5.7% and 6.6%) [24, 25], Brazil (19.7%) [26], and Malaysia (11.72%) [27], which reported lower proportions than observed in the present study, with the lowest rate recorded in China at only 1.4% [28]. On the other hand, higher proportions were identified in South Africa (34.2%) [29] and Pakistan (31%) [30]. These differences may reflect variations in tuberculosis control strategies, in the resources and infrastructure of healthcare systems, as well as in socioeconomic or epidemiological factors between countries.

The World Health Organization (WHO) recommends a minimum treatment success rate of 90% for TB [31]. Treatment success is a key indicator for evaluating the performance of TB control programs and for achieving the targets proposed by the End TB Strategy [32]. The occurrence of unfavorable outcomes compromises these goals and has been associated with increased drug resistance, greater transmissibility in household and community settings, and higher mortality and clinical complication rates among affected patients [33]. In this study, the UTO observed included treatment abandonment, death, and treatment failure. These findings are consistent with the results of a meta-analysis that included 26 studies conducted in African countries, in which most unfavorable outcomes were attributed to death and treatment abandonment [34, 35]. Similarly, a retrospective cohort conducted in Antananarivo reported comparable results [34]. These findings reflect the challenges faced by many African countries in controlling TB, particularly in settings marked by limited infrastructure [36], shortages of human and material resources, difficulties in access and continuity of care, and a high burden of coinfections and social determinants that impact treatment adherence [37].

The multivariable analysis identified that the absence of DOT and persistence of positive sputum smear at the second month were significantly associated with a higher likelihood of UTO. Conversely, clinical or radiological diagnosis and a positive smear microscopy were associated with a lower probability of UTO. These results are consistent with findings from previously published studies [24, 25, 34, 38, 39]. Based on this model, a nomogram was developed to predict OTU. It demonstrated good discriminatory ability, with an AUC of 0.832 and an accuracy of 0.849. The calibration curve indicated good agreement between predicted and observed probabilities (BS = 0.119), suggesting that the model adequately represents the data. After 1,000 bootstrap resamples, the calibration remained good (BS = 0.131). A slight deviation was observed at the extremes of the curve, falling just below the ideal line, indicating a minor overestimation of the risk of UTO. However, this deviation does not compromise the model’s predictive ability.

Previously published studies that developed nomograms to predict UTO have also demonstrated good discriminatory performance [40, 41], although many of them included only patients co-infected with HIV or those with multidrug-resistant tuberculosis [8, 15, 16, 42]. The predictive performance of our model was slightly superior to that reported in these studies, which may be partially explained by the variables included in the model. In these studies, some of the variables considered were pulmonary cavitation, pulmonary infectious diseases, and extrapulmonary disseminated TB [15]; lung lesions, sex, age, and health education [8]; anemia, comorbidities, age, treatment supporter, and marital status [16]; and lesions, treatment history, recurrent chest infections, multidrug-resistant, and extensively drug-resistant TB [42]. In contrast, the nomogram developed in the present study included patient category (WHO), DOT, diagnostic method, smear positivity at the 2nd month, smear microscopy, and adverse drug reactions. These variables may possess particular predictive value in this study population, which contributed to the slightly improved performance of our model.

The developed nomogram proved to be applicable in clinical practice as a screening tool to identify patients at higher risk of UTO and to guide more rigorous follow-up for these individuals. In addition to its good performance in the calibration curve, decision curve analysis and clinical utility analysis also confirmed the nomogram’s potential as a decision-support tool in the management of TB patients. In practice, clinicians could use the nomogram at the start of treatment to estimate each patient’s risk of UTO. However, its performance may not be generalizable to other populations, and external validation is essential before clinical implementation.

This study has some limitations. First, the data were obtained from a single health center, which, although it serves as a referral unit for the district, may limit the generalizability of the model to other institutions and regions. Second, the model was not externally validated, which underscores the need for future evaluation in larger multicenter studies and real-world clinical settings. Additionally, other relevant factors that may influence UTO, such as nutritional status, tobacco use, HIV coinfection in different populations, and socioeconomic status, should be incorporated into future versions of the model to enhance its accuracy and applicability. Despite these limitations, this study has several important strengths. It is the first study in Mozambique to apply a clinical nomogram technique, offering an innovative tool with potential practical value to support clinical decision-making. Internal validation using the bootstrap method with 1,000 resamplings confirmed the model’s potential to assist healthcare professionals in the early identification of patients at higher risk of UTO, thereby contributing to the reduction of such outcomes.

## Conclusion

The findings of this study indicate a considerable occurrence of unfavorable outcomes among patients undergoing tuberculosis treatment. Factors significantly associated with UTO included the Not receiving DOT, having been previously treated for TB, a clinical or radiological diagnosis and a positive smear microscopy result. A clinical nomogram was developed, which demonstrated good predictive performance. This tool has the potential to support early identification of patients at higher risk, guide clinical decision making, and enhance the effectiveness of TB control strategies through more targeted and individualized care.

## Supplementary Information

Below is the link to the electronic supplementary material.

Supplementary Material 1 (DOCX 92.7 KB)

## Data Availability

The datasets used and/or analyzed during the current study are available from the corresponding author upon reasonable request.
