# Supplementary material for: A Novel Clinical Nomogram for Predicting Unfavorable Tuberculosis Treatment Outcomes: A Logistic Regression Risk Model
Source: J Epidemiol Glob Health. 2026 Mar 18;16(1):35. doi: 10.1007/s44197-026-00532-z (PMC13000036; doi:10.1007/s44197-026-00532-z)
Supplement: Supplementary file 1 — Supplementary Material 1 (DOCX 92.7 KB) [file 44197_2026_532_MOESM1_ESM.docx]

**Supplementary material (S)**

**S1.** Treatment Outcome Distribution


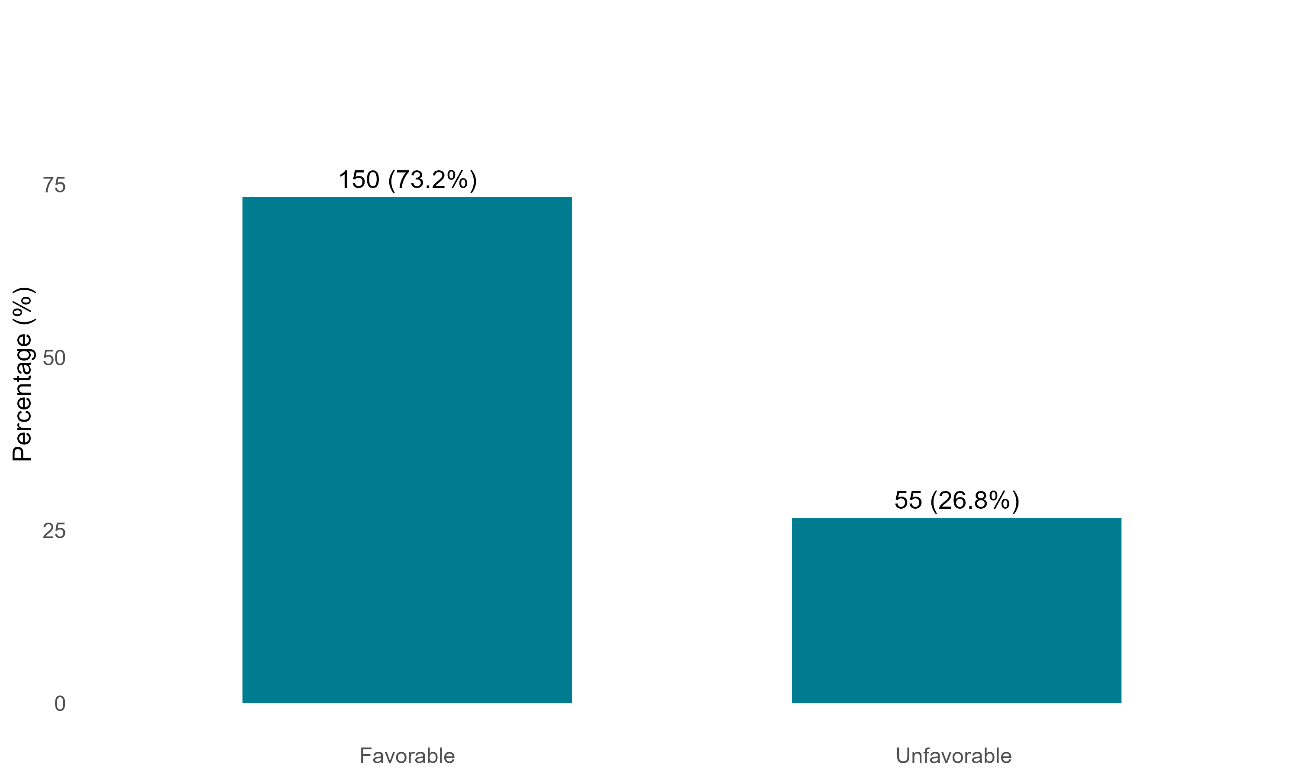


**S. Fig.1.** Treatment Outcome Distribution

**S. Table 1.** Generalized Variance Inflation Factors (GVIF) for the variables included in the logistic regression model

| **Variable** | **VIF** | **VIF Adjusted** | **AIC** |
| --- | --- | --- | --- |
| Patient category (WHO) | 1.0194 | 1.0097 | 209.19 |
| DOT | 1.0081 | 1.0041 | 210.70 |
| Diagnostic method | 1.0580 | 1.0286 | 212.68 |
| Smear microscopy | 1.0478 | 1.0236 | 218.64 |

**S. Table 2.** Hosmer–Lemeshow Goodness-of-Fit Test

| **Statistic** | **Value** |
| --- | --- |
| Test | Hosmer–Lemeshow (GOF) |
| Chi-square (χ²) | 8.47 |
| Degrees of freedom | 5 |
| p-value | 0.1323 |
| Interpretation | No evidence of lack of fit (p > 0.05) |

**S. Table 3.** Point assignment and predicted probability of UTO

| **Variable** | **Category** | **Points** |
| --- | --- | --- |
| **WHO TB Category** | New case | 0 |
|  | Previously treated | 31 |
| **DOT Status** | Under direct observation | 0 |
|  | Not observed | 34 |
| **Diagnosis Method** | Bacteriological | 100 |
|  | Clinical/X-ray | 0 |
| **Smear Positivity (2nd month)** | No | 0 |
|  | Not applicable | 51 |
|  | Yes | 64 |
| **Smear Microscopy** | No | 22 |
|  | Yes | 0 |
| **Adverse Drug Reactions** | No | 0 |
|  | Yes | 87 |
| **Total Points** | **Predicted Probability of UTO** |  |
| 100 - 123 | 0.05 – 0.10 |  |
| 148 - 165 | 0.20 – 0.30 |  |
| 179 - 191 | 0.40 – 0.50 |  |
| 204 | 0.60 |  |
| 218 | 0.70 |  |
| 235 | 0.80 |  |
| 260 | 0.90 |  |
| 283 | 0.95 |  |
| 335 | 0.99 |  |

**S. Table 4.** Classification performance metrics at the optimal Youden threshold (0.25)

| **Metric** | **Value** |
| --- | --- |
| Accuracy (IC 95%) | 0. 824 (0.765; 0.874) |
| No Information Rate | 0.7317 |
| P-value (Acc > NIR) | 0.0012 |
| Kappa | 0.5578 |
| McNemar’s Test (p-value) | 0.868 |
| Sensitivity | 0.6909 |
| Specificity | 0.8733 |
| Positive Predictive Value (PPV) | 0.6667 |
| Negative Predictive Value (NPV) | 0.8851 |
| Prevalence | 0.2683 |
| Detection Rate | 0.1854 |
| Detection Prevalence | 0.2780 |
| Balanced Accuracy | 0.7821 |
| Positive Class | 1 |
| Confusion Matrix | True Negative (TN) 130  False Positive (FP) 20  False Negative (FN) 17  True Positive (TP) 38 |

**S. Table 5.** Model Performance Statistics

| Component | Performance | Value |
| --- | --- | --- |
| Model Likelihood Ratio Test | LR χ² | 77.61 |
|  | Degrees of freedom | 7 |
|  | Pr(>χ²) | <0.0001 |
|  | max \|deriv\| | 7e-08 |
| Discrimination Indexes | C-index (Harrell’s C) | 0.832 |
|  | Dxy | 0.663 |
|  | Gamma | 0.698 |
|  | Tau-a | 0.262 |
| Rank Discrimination | R² (Nagelkerke) | 0.291 |
|  | R² (adjusted, 7; 205) | 0.458 |
|  | R² (adjusted, 7; 120.7) | 0.443 |
| Calibration | Brier Score | 0.119 |

**S. Table 6.** Internal Validation via Bootstrapping (1,000 resamples)

| **Index** | **Original** | **Training** | **Test** | **Optimism** | **Optimism-Corrected** |
| --- | --- | --- | --- | --- | --- |
| Dxy | 0.6632 | 0.6848 | 0.6516 | 0.0332 | 0.6300 |
| R² | 0.4585 | 0.4870 | 0.3976 | 0.0894 | 0.3691 |
| Intercept | 0.0000 | 0.0000 | –0.2387 | 0.2387 | –0.2387 |
| Slope | 1.0000 | 1.0000 | 0.7560 | 0.2440 | 0.7560 |
| Emax | 0.0000 | 0.0000 | 0.1080 | 0.1080 | 0.1080 |
| D | 0.3737 | 0.4046 | 0.3155 | 0.0891 | 0.2846 |
| U | –0.0098 | –0.0098 | 0.0325 | –0.0423 | 0.0325 |
| Q | 0.3835 | 0.4143 | 0.2830 | 0.1314 | 0.2521 |
| Brier Score | 0.1192 | 0.1142 | 0.1255 | –0.0113 | 0.1305 |
| g | 1.8804 | 2.7831 | 1.9057 | 0.8774 | 1.0030 |
| gp | 0.2781 | 0.2825 | 0.2500 | 0.0325 | 0.2456 |

**S. Table 7**. Model Discrimination (C-Index/AUC)

| **Metric** | **C-Index**/AUC |
| --- | --- |
| Original C-index/AUC | 0.8316 |
| Training C-index/AUC | 0.8424 |
| Test C-index/AUC | 0.8258 |
| Optimism-corrected C-index/AUC | 0.8150 |


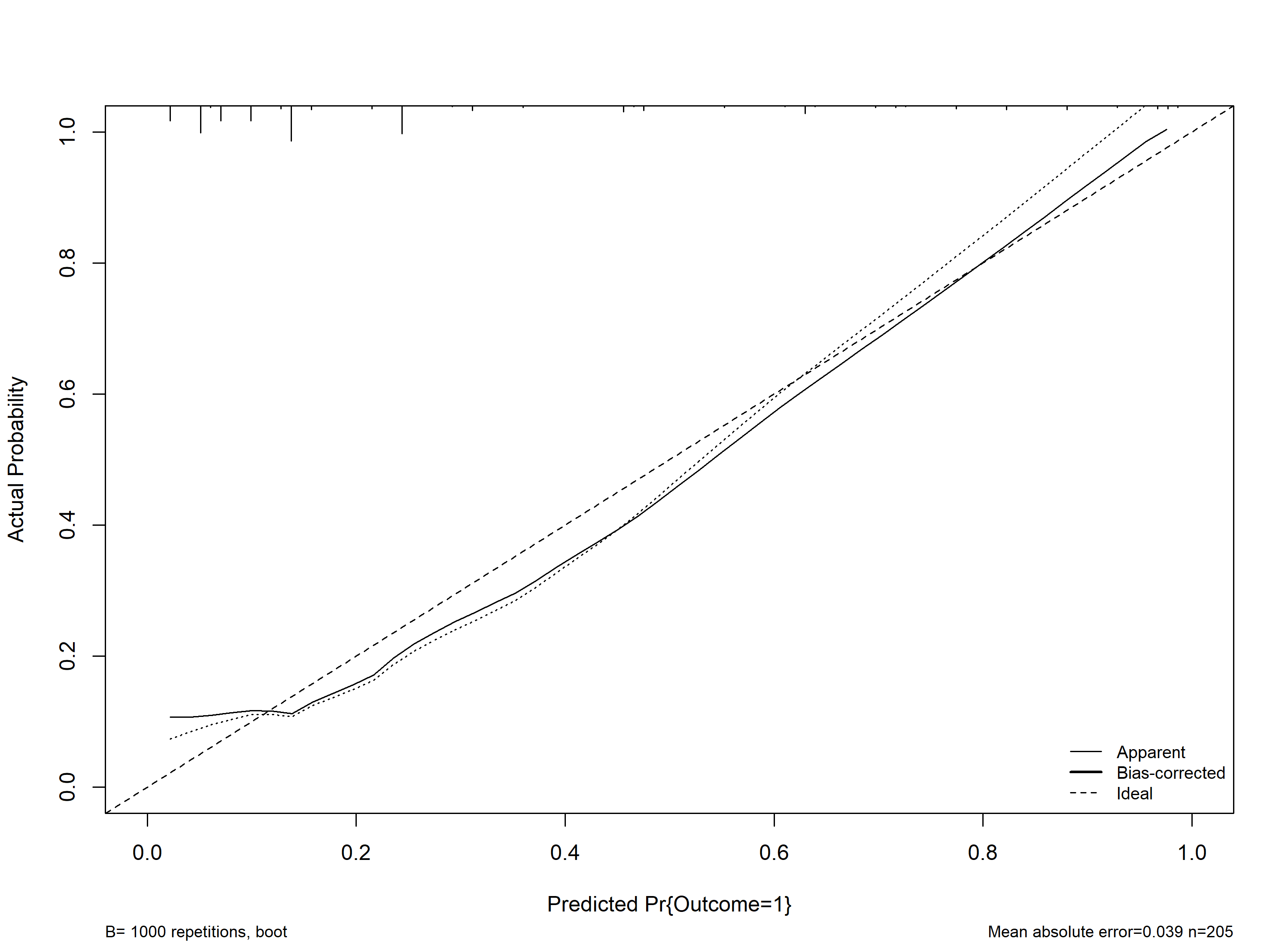


**S. Fig.2.** Calibration plot for the predictive nomogram model.
